# Dr. William H. Myers

**Published:** 1907-03

**Authors:** 


Dr. William H. Myers, of Fort Wayne, Ind., died at his home January 5, 1907, after an illness of two weeks, aged 80 years. Dr. Myers was a graduate of Jefferson Medical College, 1855; served as surgeon of the 30th Indiana volunteer infantry during the civil war; was a leader in the establishment of hospitals at Fort Wayne, and in the organisation of the Fort Wayne Medical College; was a surgeon of distinction and was noted in addition for his capability as an expert medical witness. He was a Founder of the American Association of Obstetricians and Gynecologists, becoming an Honorary Fellow in later years.

